# Metabolomics analysis delineates the therapeutic effects of Yinlan Tiaozhi capsule on triton WR-1339 -induced hyperlipidemia in mice

**DOI:** 10.3389/fphar.2023.1252146

**Published:** 2023-10-30

**Authors:** Guanlin Xiao, Aili Xu, Jieyi Jiang, Zhao Chen, Yangxue Li, Sumei Li, Weitao Chen, Jingnian Zhang, Canchao Jia, Zhihao Zeng, Xiaoli Bi

**Affiliations:** ^1^ Guangdong Province Engineering Technology Research Institute of Traditional Chinese Medicine/Guangdong Provincial Key Laboratory of Research and Development in Traditional Chinese Medicine, Guangzhou, China; ^2^ School of the Fifth Clinical Medicine, Guangzhou University of Chinese Medicine, Guangzhou, China

**Keywords:** Yinlan Tiaozhi capsule, hyperlipidemia, metabolomics, inflammation, angiogenesis

## Abstract

Hyperlipidemia is a disorder of lipid metabolism resulting from abnormal blood lipid metabolism and is one of the most frequent metabolic diseases that endanger people’s health. Yinlan Tiaozhi capsule (YL) is a formulated TCM widely used to treat hyperlipidemia. The purpose of this study was to discover biomarkers utilizing untargeted metabolomics techniques, as well as to analyze the mechanisms underlying the changes in metabolic pathways linked to lipid-lowering, anti-inflammation, and regulation of angiogenesis in hyperlipidemia mice. To assess the efficacy of YL, serum total cholesterol (TC), triglycerides (TG), low-density lipoprotein cholesterol (LDL-c), and high-density lipoprotein cholesterol (HDL-c) levels were measured. Biochemical examinations showed that YL significantly reduced the levels of TC, TG, LDL-c, *Il6*, *Tnf-α*, and *Vegfa* in hyperlipidemia mice (*p* < 0.01). YL also significantly increased the levels of HDL-c and *Alb* (*p* < 0.01). Twenty-seven potential serum biomarkers associated with hyperlipidemia were determined. These differential metabolites were related to the reduction of serum lipid levels in hyperlipidemia mice, probably through metabolic pathways such as linoleic acid metabolism, glycerophospholipid metabolism, phenylalanine metabolism, phenylalanine, tyrosine and tryptophan biosynthesis, and D-glutamine and D-glutamate metabolism. Further correlation analysis showed that the serum lipid reduction through YL was related to the metabolites (amino acid metabolites, phospholipids metabolites, and fatty acids metabolites). The present study reveals that YL has a profound effect on alleviating triton WR-1339-induced hyperlipidemia, inflammation, and angiogenesis and that the positive effects of YL were primarily associated with the correction of metabolic abnormalities and the maintenance of metabolite dynamic balance.

## 1 Introduction

Hyperlipidemia is a metabolic disorder characterized by abnormally increased levels of plasma total cholesterol (TC), triglycerides (TG), and low-density lipoprotein-cholesterol (LDL-C), accompanied by decreased levels of high-density lipoprotein-cholesterol (HDL-c) (Birger et al., 2021). Hyperlipidemia is a primary reason for many cardiovascular diseases and is closely correlated with diabetes, obesity, and atherosclerosis (Jia et al., 2021). Pharmacological interventions are the main therapy for hyperlipidemia, and the main drugs include statins, fibrates, and cholesterol absorption inhibitors. However, most medications cause adverse effects such as gastrointestinal problems (Tacherfiout et al., 2018). Presently, an appropriate way to alleviate dyslipidemia and avoid adverse effects may be through the use of natural medicines, and the efficacy of TCM in treating hyperlipidemia has been increasingly investigated (Tong et al., 2018).

Yinlan Tiaozhi capsule (YL) is a formulated traditional Chinese medicine (TCM) comprising Citri Grandis Exocarpium, Ginkgo Folium, Gynostemma pentaphyllum, and propolis. It has been clinically used for the treatment of hyperlipidemia and is presently in the phase II clinical stage (2012L01011) (Chen et al., 2019; Xiao et al., 2023a; Xiao et al., 2023b). Previously, the chemical constituents of YL were investigated using UPLC-Q-TOF-MS/MS, and an overall of 66 compounds were identified, which mainly included flavonoids, saponins, lactones, and organic acids. The primary components of YL, such as naringenin and ferulic acid, may treat hyperlipidemia by modulating angiogenic mechanisms and suppressing the inflammatory response, according to network pharmacology integrated with molecular docking and experimental verification (Xiao et al., 2023a). Meanwhile, preliminary data showed that the anti-hyperlipidemia actions of YL may be associated with the inhibition of PXR expression, promotion of bile acid excretion, TG hydrolysis, and RCT processes(Xiao et al., 2023a). YL contains multiple active ingredients and achieves its pharmacological effects through multiple pathways and targets. Consequently, it is crucial to understand the integrated mechanism of the overall metabolism of YL under conditions of hyperlipidemia.

Metabolomics, with its systematic perspective and strategy, has been applied to comprehensively characterize and quantify metabolite levels and is the optimum instrument to illuminate the safety and efficacy of TCM, the pathophysiology of diseases, and the mechanism of drug action (Su et al., 2019; Wu et al., 2019; Zhao et al., 2021). Metabolomics is a “top-down” approach to defining the overall alterations in living systems at the metabolic level for complex metabolic diseases (Sun et al., 2012). This study performed serum biochemical analysis, metabolic pathway analysis, and analysis of metabolic changes to identify the underlying biomarkers of hyperlipidemia and to investigate the mechanisms and pathways of lipid-lowering of YL in hyperlipidemia mice.

## 2 Materials and methods

### 2.1 Materials and animals

The YL (Batch number: 20220801) was obtained from Guangdong Efong Pharmaceutical Co. Ltd. (Foshan, China). Triton WR-1339 was purchased from Sigma-Aldrich (Shanghai, China). The chemical profile of YL analyzed by UPLC-QTOF-MS/MS is shown in Supplementary Figure S1. The 24 male Kunming (KM) mice (18–22 g body weight) were obtained from the Guangdong Medical Laboratory Animal Center (Guangzhou, China) (Permit number: 44007200106576). All animal experiments were approved by the Guangdong Provincial Engineering Technology Institute of Traditional Chinese Medicine (Guangzhou, China).

### 2.2 Animal administration

**FIGURE 1:**
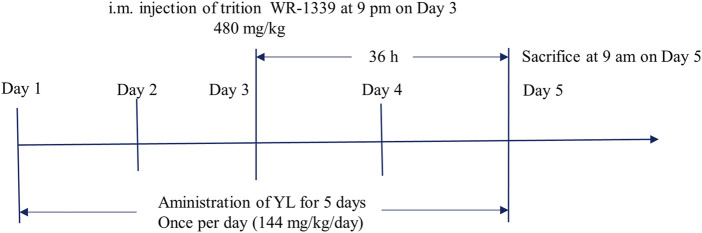
Process of administration of YL and hyperlipidemia modeling.

Combined with the group’s previous research and clinical trials (Chen et al., 2019; Li et al., 2019; Li et al., 2020; Xiao et al., 2023a), the animal experimental process is shown in Figure 1, and the experimental program is specified as follows: The 24 male KM mice were raised in a barrier system under standard room temperature 24°C ± 2°C with the humidity of 70% ± 5%. The mice were subjected to 12 h light/dark cycle conditions and fed with normal food and water. The experiment mice were randomly divided into the following 4 groups of 6 mice in each group: control group (CG), model group (MG), fenofibrate group (26 mg/kg) (Fenofibrate) (Li et al., 2020), and YL group (144 mg/kg) (YL) (Chen et al., 2019). The administration groups were given corresponding drugs by gavage and once a day for 5 days. On the third day of administration, all groups excluding the CG were administered triton WR-1339 (480 mg/kg) intramuscularly to build an acute hyperlipidemia model. On the fifth day, after gavage administration for 1 hour, all mice were anesthetized with isoflurane and sacrificed through inner canthus artery exsanguination. Blood samples were preserved at room temperature for 30 min and centrifuged at 3,000 rpm at 4°C for 15 min to isolate the serum. Liver tissue was surgically extracted from each mouse. The wet weights of the organs were recorded and the tissues were saved at −80°C until further experiments.

### 2.3 Biochemical analysis

The levels of TC, TG, HDL-c, and LDL-c in serum were determined by using commercial kits (Nanjing Jiancheng Bioengineering Institute, China) according to the manufacturer’s instructions.

### 2.4 Histopathology

The liver tissues of mice were fixed with 4% paraformaldehyde solution, then the samples were dehydrated, embedded in paraffin, and sliced into 4 μm thick sections for H&E staining, and the histomorphology of the livers was observed and recorded using a microphotographic operating system.

### 2.5 Sample preparation

Serum metabolomic analysis was conducted on all mice in each group as individual differences in each group were taken into account. Serum samples were collected and stored at −80°C. Prior to analysis, the serum samples were defrosted at room temperature, and 100 μL serum was added to 400 μL of pre-cooled acetonitrile and water mixture (1:1, v/v) fixed in a 1.5 mL microtubes, placed in −20°C refrigerator for 1 h, centrifuge for 15 min (4°C, 12,000 rpm), transfer the supernatant (400 μL) to a new 1.5 mL centrifuge tube, and blow dry with nitrogen. Add 100 μL of pre-cooled acetonitrile and methanol mixture (1:1, v/v), vortex for the 30 s, centrifuge for 15 min (4°C, 12,000 rpm), and accurately absorb 80 uL of the supernatant into the injection bottle for sample analysis. Quality control (QC) sample was obtained by mixing 10 μL of serum samples from the above mice, and the sample was prepared as described above. The QC samples were injected at 6 intervals of study samples to assess the stability of the analytical system (Ren et al., 2023).

### 2.6 UPLC-Q-TOF/MS analysis

The UPLC analysis was carried out on a SHIMADZU ExionLC system (Shimadzu, Japan). The chromatographic separations were achieved on a Waters UPLC BEH C18 column (2.1 mm × 100 mm, 1.7 μm), flow rate 0.3 mL min^−1^, injector temperature 4°C, column temperature 35°C, injection volume 1 μL. Mobile phase: gradient elution of acetonitrile (A) −0.1% formic acid water (B) (0–1 min, 2%A; 1–3 min, 2%–10%A; 3–7 min, 10%–40%A; 7–16 min, 40%–75%A; 16–20 min, 75%-98%A; 20–23 min, 98%A).

The high-resolution mass detection was performed on an AB SCIEX X500R QTOF-MS/MS system (Sciex, United States). MS was performed both in positive and negative ion modes with electrospray ionization (ESI). The optimization source parameters were set as follows: ion voltage: −4,500 V and + 5,500 V, Gas1: 55 psi; Gas2: 55 psi; curtain gas: 35 psi; de-clustering potential voltage: 60 V; ion source temperature: 500°C; collision energy: 35 V; collision energy spread: 15 V; full scan: *m*/*z* 50–1,000.

### 2.7 Data processing and multivariate analysis

Multivariate statistical analysis was conducted using SIMCA 14.1 software with unsupervised principal component analysis (PCA) and orthogonal partial least discriminant analysis (OPLS-DA) models, and the results of OPLS-DA needed to be further confirmed by 200 times permutation evaluations. Statistical analyses were then performed using the online tool Metaboanalyst 5.0 for univariate student *t*-test (*t*-test) and finally for difference multiple (FC value) analysis. The metabolites with significantly different (Variable important in projection (VIP) > 1, *p* < 0.05, Fold change (FC) value < 0.8, or FC value > 1.2) between MG and CG and between YL and MG were screened for preliminary identification and analysis. Further analysis of these metabolites was performed to screen for metabolites that were significant differences between YL and MG and showed a trend of recovery compared with CG were screened as the differential metabolites for treating hyperlipidemia. Potential biomarkers were characterized according to the METLIN and Human Metabolome Database databases, and pathways of differential metabolites were analyzed using the online software MetaboAnalyst 5.0 (http://www.metaboanalyst.ca/) (Shi et al., 2023).

### 2.8 Quantitative RT-PCR

Approximately 50 mg of liver were transferred to a 1.5 mL grinding tube. Liver samples were acquired by mechanically homogenized under an ice water bath and centrifuged at 12,000 rpm for 10 min at 4°C, and the supernatant was subsequently collected. Extraction of total RNA from liver tissues was performed using Trizol reagent (Dingguo Changsheng, Beijing, China), and reverse transcription was conducted. RT-qPCR reactions were conducted on an IQ™5 RT-Q-PCR system (Bio-Rad, Hercules, California, United States) with SYBR Green detection. The sequences of primers are shown in Table 1.

**TABLE 1:** The primer sequence formation.

Gene name	Forward primer (5′-3′)	Reverse primer (5′-3′)
*Il6*	AGT​TGT​GCA​ATG​GCA​ATT​CTG​A	CTC​TGA​AGG​ACT​CTG​GCT​TTG​TC
*Tnf-α*	CCC​TCA​CAC​TCA​CAA​ACC​ACC	CTT​TGA​GAT​CCA​TGC​CGT​TG
*Alb*	AAC​AAG​AGC​CCG​AAA​GAA​ACG	CTG​GCA​ACT​TCA​TGC​AAA​TAG​TG
*Vegfa*	GTA​ACG​ATG​AAG​CCC​TGG​AGT​G	TCA​CAG​TGA​ACG​CTC​CAG​GAT
*Gapdh*	CCT​CGT​CCC​GTA​GAC​AAA​ATG	TGA​GGT​CAA​TGA​AGG​GGT​CGT

### 2.9 Statistical analysis

SPSS software was utilized to analyze the data by one-way ANOVA and all values were presented as means ± SD. *p* < 0.05 was considered statistically significant. Metabolomics data are normalized by MetaboAnalyst 5.0 and exported into SIMCA-P 14.1 software for processing (Shi et al., 2023).

## 3 Results

### 3.1 Effect of YL in the treatment of triton WR-1339-induced hyperlipidemia in mice

**FIGURE 2:**
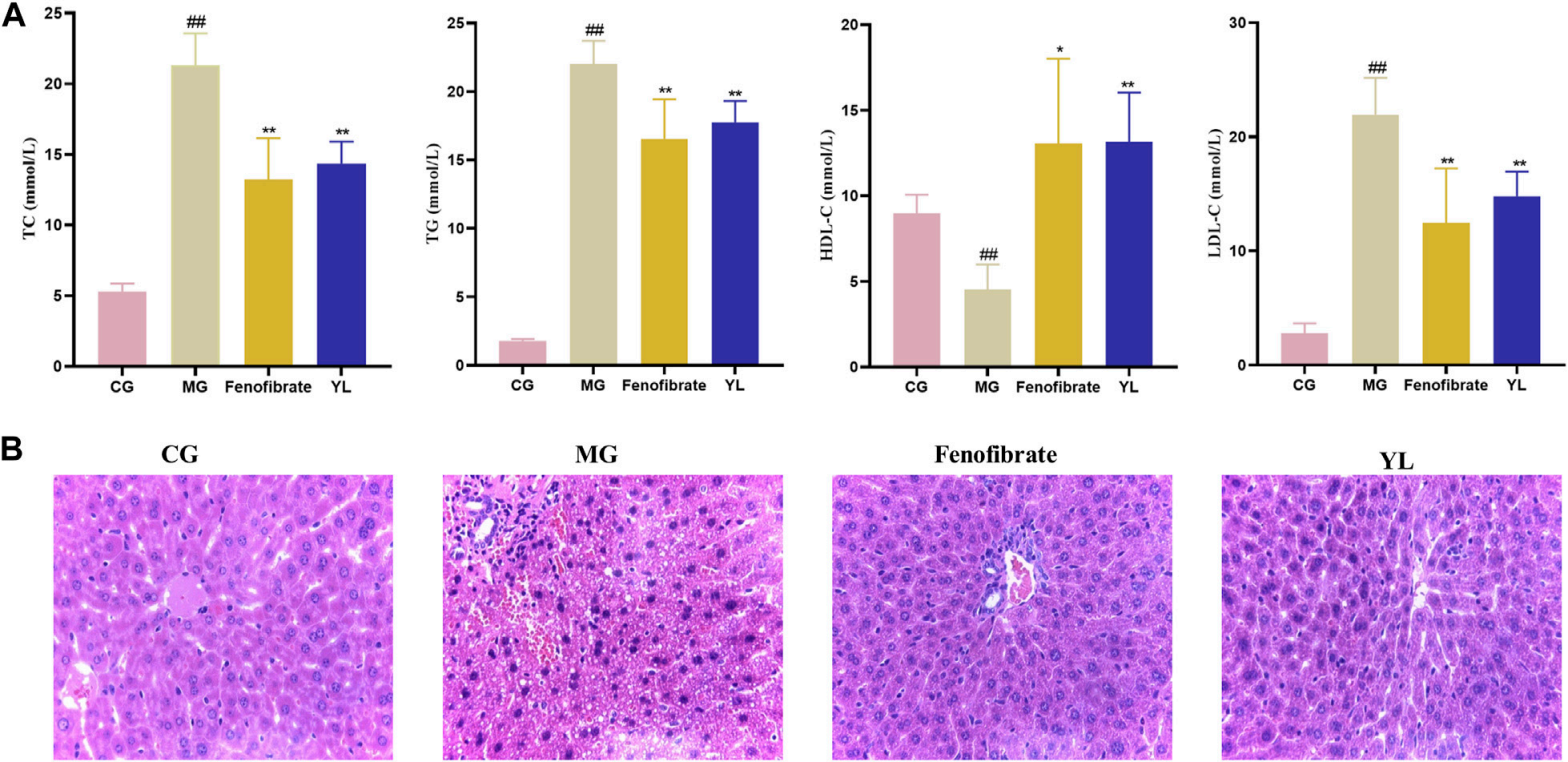
Effect of YL on serum and liver in triton WR-1339-induced hyperlipidemia mice. **(A)** Biochemical analyses of serum TC, TG, HDL-c and LDL-c. Values are mean ± SD, *n* = 6. ^#^ *p* < 0.05, ^##^ *p* < 0.01 vs CG; *p < 0.05, ^**^ *p* < 0.01 vs MG. **(B)** H&E staining of liver tissue (×40 magnification).

As shown in Figure 2A, serum TC, TG, and LDL-c levels were significantly higher (all *p* < 0.01), and the serum HDL-c levels were lower (*p* < 0.01) in the MG compared with mice in the CG. After administration of YL or Fenofibrate, the serum levels of TC, TG, and LDL-c (all *p* < 0.01) were decreased and the serum HDL-c (*p* < 0.05, *p* < 0.01) levels were increased in mice compared to the MG. These results suggested that YL effectively ameliorated dyslipidemia in hyperlipidemia mice induced by triton WR-1339.

The histologic data were consistent with observations of serum lipid levels in mice. The liver cells of mice in the CG were neatly arranged, with intact nuclei and no inflammatory cell infiltration or vacuolar lesions. The liver cells of mice in the MG were disordered, with unclear cell boundaries, absence of some nuclei, diffuse steatosis of liver cells, and increased vacuolar lesions and inflammatory infiltration. Compared with the MG, the liver cells of the Fenofibrate were neatly arranged, the number of fat vacuoles was significantly reduced, and the cell morphology was improved, but there was still inflammatory infiltration; the liver cell morphology of the YL was significantly improved, there was no inflammatory infiltration, and the scope of the lesions was significantly reduced (Figure 2B).

### 3.2 Quality evaluation of metabolomics data

**FIGURE 3:**
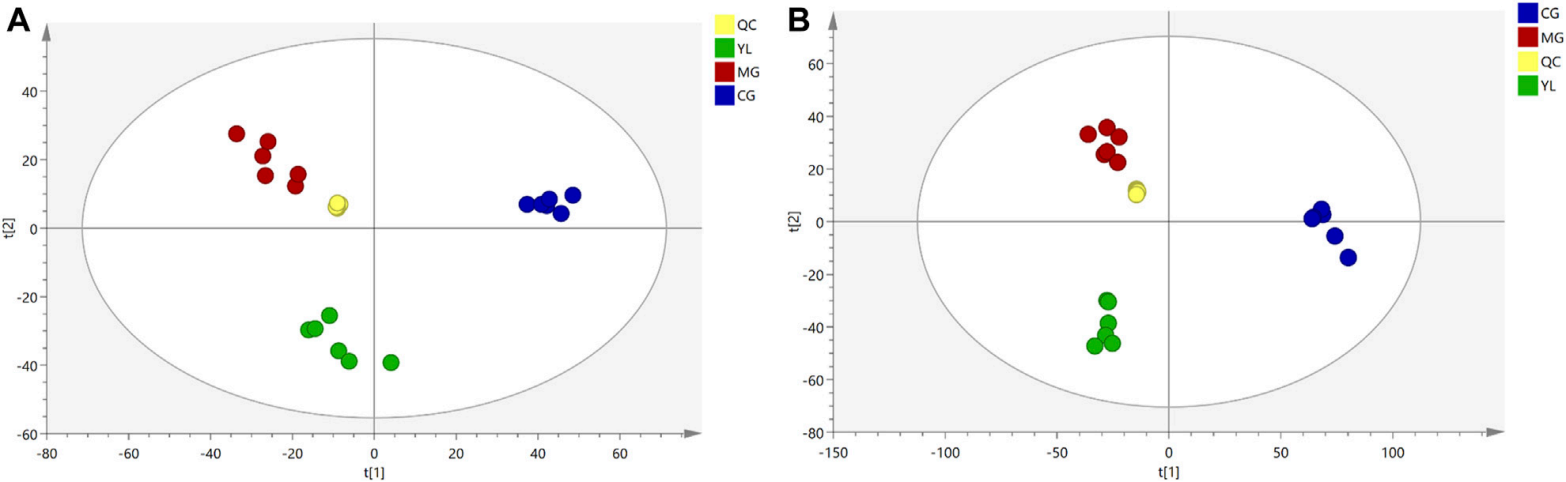
PCA for serum from CG, MG, and YL in the negative **(A)** and positive **(B)** ESI mode.

As illustrated in Figure 3, the QC samples displayed good clustering in the PCA score plot, demonstrating that the pre-processing and experimental conditions of the samples were reliable and the data obtained were accurate.

### 3.3 YL-modulated serum metabolomic profiling

**FIGURE 4:**
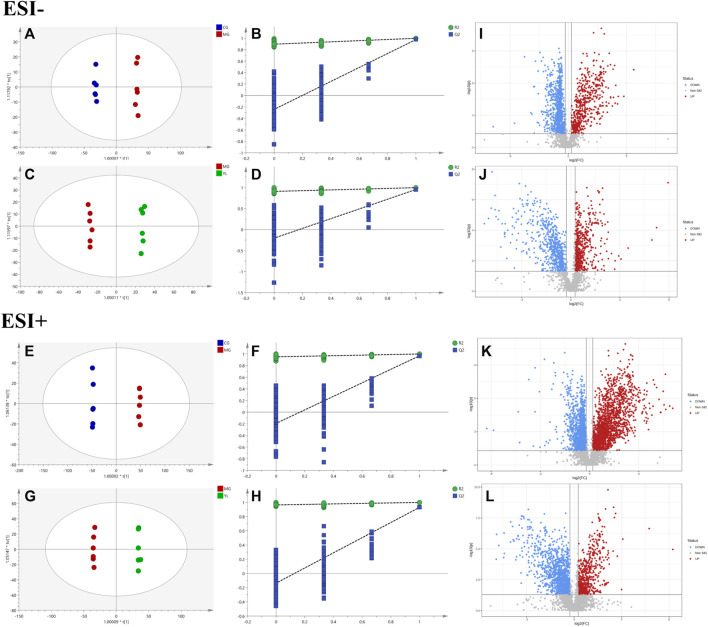
YL intervention on hyperlipidemia mice. Negative model: **(A)** Score plot of OPLS-DA between MG and CG. **(B)** The result of cross-validation of OPLS-DA model between MG and CG. **(C)** Score plot of OPLS-DA between YL and MG. **(D)** The result of cross-validation of OPLS-DA model between YL and MG. Positive model: **(E)** Score plot of OPLS-DA between MG and CG. **(F)** The result of cross-validation of OPLS-DA model between MG and CG. **(G)** Score plot of OPLS-DA between YL and MG. **(H)** The result of cross-validation of OPLS-DA model between YL and MG. Negative model: **(I)** The volcano plot of the differential metabolites was screened in the MG and CG. **(J)** The volcano plot of the differential metabolites was screened in the YL and MG. Positive model: **(K)** The volcano plot of the differential metabolites was screened in the MG and CG. **(L)** The volcano plot of the differential metabolites was screened in the YL and MG.

The PCA results indicated a significant separation of the three groups in positive and negative ion modes (Figure 3). The results indicated that the serum biochemical of hyperlipidemia mice was disordered and the metabolic pattern was significantly altered after oral administration of YL. OPLS-DA models were performed between the CG and MG and the MG and YL, respectively. The results of the OPLS-DA score plot and the 200 permutation test in serum metabolomics were presented in Figures 4A–H. The R^2^Y, Q^2^, and CV-ANOVA (*p*-values) of the OPLS-DA model for serum metabolomics were presented in Table 2. The data in the table revealed that the R^2^Y values of each model were greater than 0.997 and the Q^2^ values were greater than 0.928, indicating that the classification and explanation ability of the models was excellent (Gao et al., 2023). The 200 times permutation tests suggested that the OPLS-DA models established were all reliable and not overfitting (Yang et al., 2020). VIP, P, and FC values were visualized by volcano plot for selecting differential metabolites (Figures 4I–L). 27 differential metabolites were characterized with a VIP >1 and *p* < 0.05 (FC > 1.2 or <0.8) between MG vs CG or YL vs. MG (Table 3).

**TABLE 2:** The parameters of the OPLS-DA model.

Mode	Group	R^2^X	R^2^Y	Q^2^	CV-ANOVA
Positive	MG vs. CG	0.558	0.999	0.968	2.51e^−5^
	YL vs. MG	0.427	0.999	0.929	0.0004
Negative	MG vs. CG	0.596	0.998	0.976	9.29e^−6^
	YL vs. MG	0.535	0.998	0.954	8.85e^−5^

**TABLE 3:** Identification and change trend of potential biomarkers.

Ion mode	HMDB ID	Metabolites	Formula	RT (min)	MG vs. CG	YL vs. MG
ESI^-^	HMDB0000190	L-Lactic acid	C_3_H_6_O_3_	1.27	↓^##^	↑**
	HMDB0000148	L-Glutamic acid	C_5_H_9_NO_4_	1.47	↓^##^	↑**
	HMDB0000159	L-Phenylalanine	C_9_H_11_NO_2_	4.02	↓^##^	↑**
	HMDB0000714	Hippuric acid	C_9_H_9_NO_3_	5.80	↓^##^	↑**
	HMDB0000826	Pentadecanoic acid	C_15_H_30_O_2_	19.80	↓^##^	↑**
	HMDB0000673	Linoleic acid	C_18_H_32_O_2_	18.44	↑^##^	↓**
	HMDB0000573	Elaidic acid	C_18_H_34_O_2_	19.58	↑^##^	↓**
	HMDB0004669	9-OxoODE	C_18_H_30_O_3_	15.53	↑^##^	↓**
	HMDB0004702	12,13-EpOME	C_18_H_32_O_3_	16.03	↑^##^	↓**
	HMDB0007855	LysoPA(18:1(9Z)/0:0)	C_23_H_46_NO_7_P	15.32	↓^##^	↑**
ESI^+^	HMDB0012497	1-Pyrroline	C_4_H_7_N	1.04	↓^##^	↑**
	HMDB0000097	Choline	C_5_H_13_NO	0.91	↓^##^	↑**
	HMDB0003229	Palmitoleic acid	C_16_H_30_O_2_	19.14	↑^##^	↓**
	HMDB0000220	Palmitic acid	C_16_H_32_O_2_	19.15	↑^##^	↓**
	HMDB0031934	(9S,10E,12Z,15Z)-9-Hydroxy-10,12,15-octadecatrienoic acid	C_18_H_30_O_3_	15.51	↑^##^	↓**
	HMDB0001388	Alpha-Linolenic acid	C_18_H_30_O_3_	16.00	↓^##^	↑**
	HMDB0003759	5a-Pregnane-3,20-dione	C_21_H_32_O_2_	18.87	↑^##^	↓**
	HMDB0000253	Pregnenolone	C_21_H_32_O_2_	17.94	↑^##^	↓**
	HMDB0002395	Ursolic acid	C_30_H_48_O_3_	14.09	↓^##^	↑**
	HMDB0011473	LysoPE(0:0/16:0)	C_21_H_44_NO_7_P	15.19	↓^##^	↑**
	HMDB0002815	LysoPC(18:1(9Z))	C_26_H_52_NO_7_P	15.37	↓^##^	↑**
	HMDB0010386	LysoPC(18:2(9Z,12Z))	C_26_H_50_NO_7_P	13.23	↓^##^	↑**
	HMDB0010395	LysoPC(20:4(5Z,8Z,11Z,14Z))	C_28_H_50_NO_7_P	14.33	↓^##^	↑**
	HMDB0010384	LysoPC(18:0)	C_26_H_54_NO_7_P	14.76	↓^##^	↑**
	HMDB0010393	LysoPC(20:3(5Z,8Z,11Z))	C_28_H_52_NO_7_P	15.12	↓^##^	↑**
	HMDB0010392	LysoPC(20:2(11Z,14Z))	C_28_H_54_NO_7_P	14.77	↓^##^	↑**
	HMDB0010391	LysoPC(20:1(11Z))	C_28_H_56_NO_7_P	15.97	↓^##^	↑**

^#^ *p* < 0.05, and ^##^ *p* < 0.01, MG, vs. CG.

*p < 0.05, and **p < 0.01, YL, vs. MG., the structures, molecular weights, and codes of differential metabolites were assigned according to the human metabolome database and KEGG, compound database.

### 3.4 YL regulated metabolomic pathways in the hyperlipidemia mice

**FIGURE 5:**
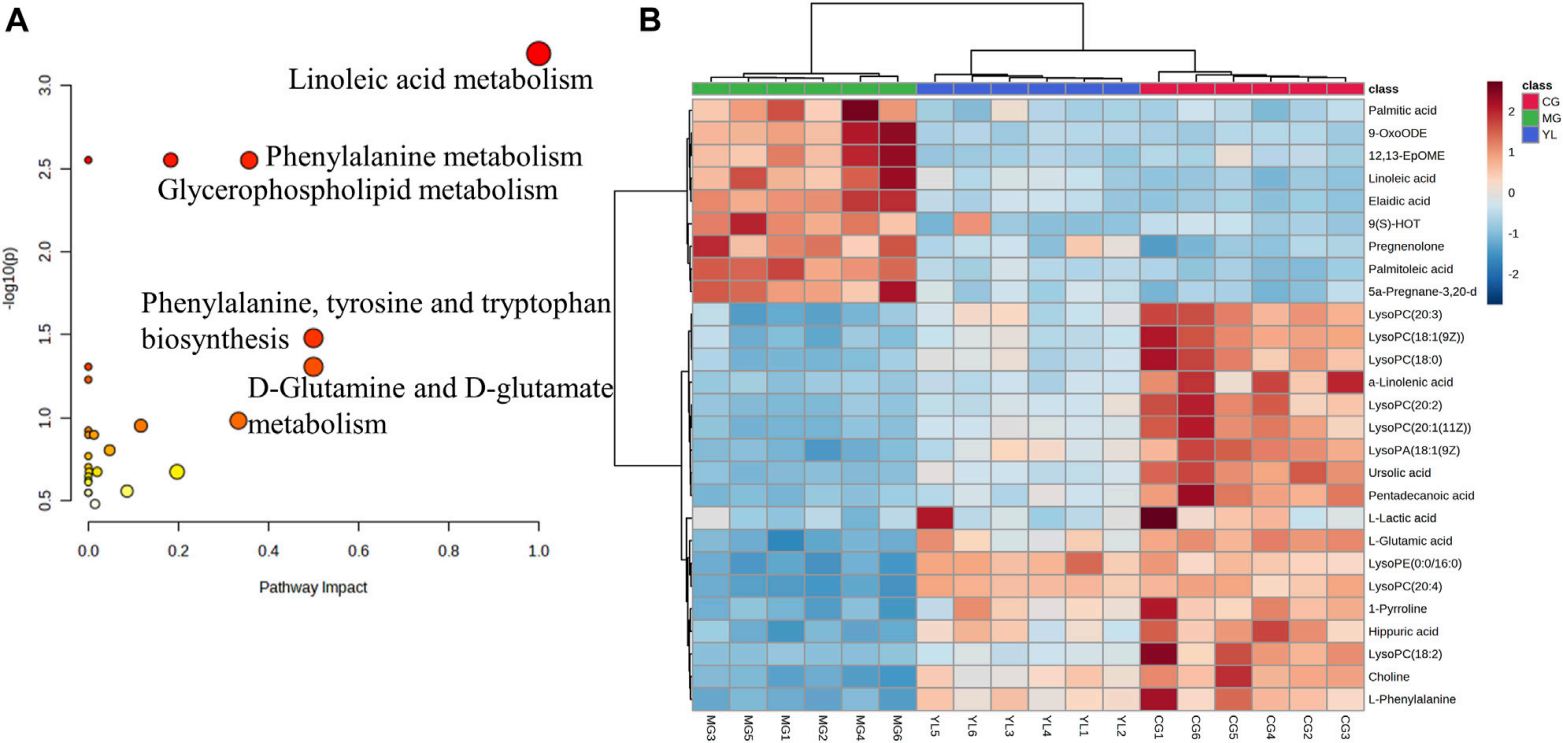
Metabolic pathway analysis according to the identified metabolites associated with hyperlipidemia. **(A)** Heatmap to visualize the abundance of biomarkers in each group. **(B)** Hierarchical clustering heatmap of the 27 differential metabolites with the degree of variation marked in red (upregulation) and blue (downregulation).

**FIGURE 6:**
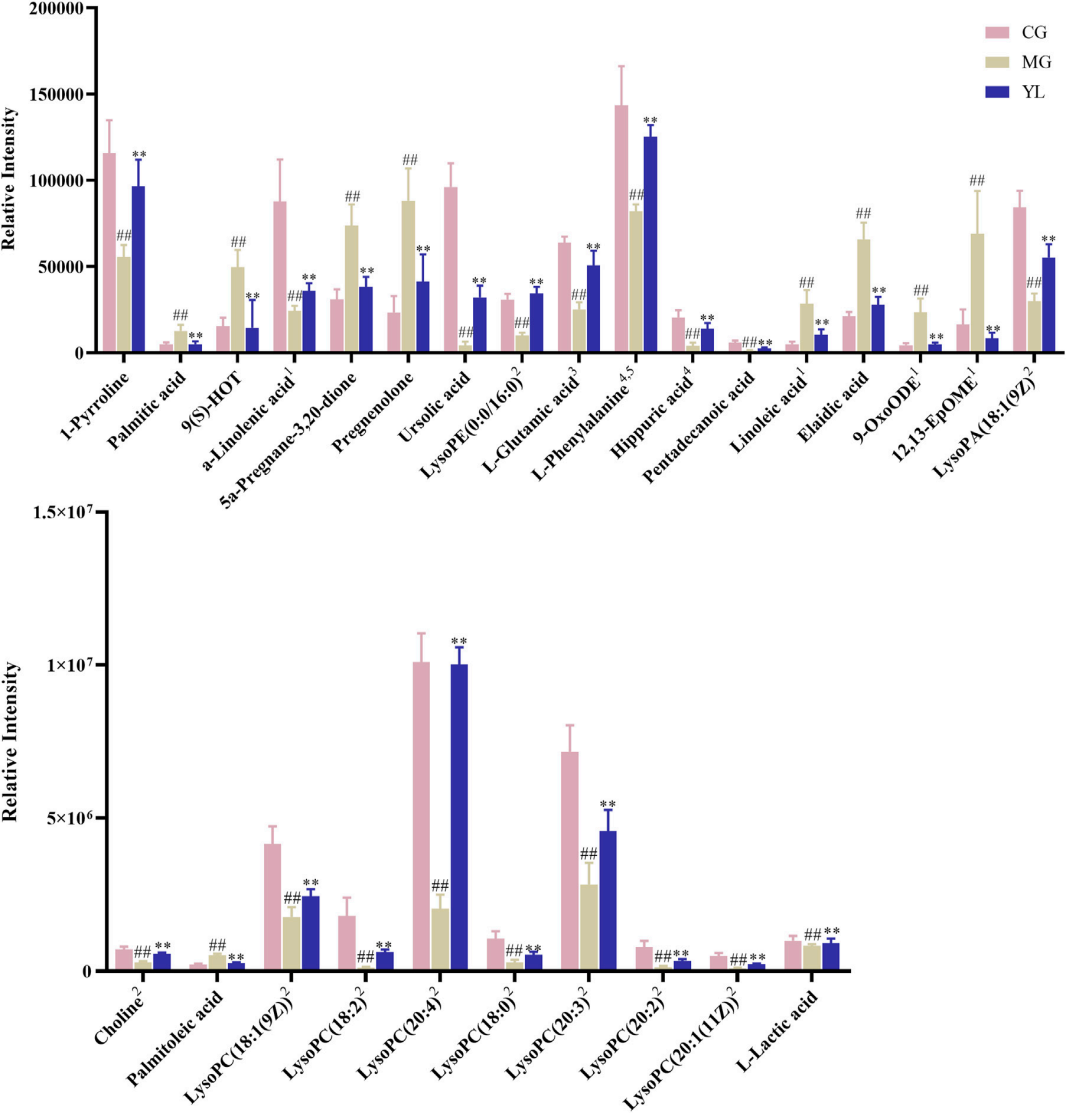
The relative contents of potential serum biomarkers in each groups. (Compared with CG, ^#^ *p* < 0.05, ^##^ *p* < 0.01; compared with MG, ^*^ *p* < 0.05, ^**^ *p* < 0.01), ^1^Linoleic acid metabolism, ^2^Glycerophospholipid metabolism, ^3^D-Glutamine and D-glutamate metabolism, ^4^Phenylalanine metabolism, ^5^Phenylalanine, tyrosine and tryptophan biosynthesis.

To obtain a deeper understanding of the molecular mechanisms of YL, pathway analysis was performed on 27 differential metabolites. Metabolite data were imported into pathway analysis to investigate metabolic pathway weights, enriching 26 metabolic pathways, five of which were highly emphasized with raw *p* < 0.05 or pathway impact >0.05 (Sun et al., 2022). They were linoleic acid metabolism, glycerophospholipid metabolism, phenylalanine metabolism, Phenylalanine, tyrosine and tryptophan biosynthesis, and D-Glutamine and D-glutamate metabolism (Figure 5A). As indicated in Figure 5B, the clustering analysis of the heat map for all metabolites showed the differences in relative levels between the three groups. In addition, the heat map was also employed to show the relative abundance of the different metabolites in each sample. Compared with the CG, 9 metabolites were upregulated significantly in the MG, including 9-OxoODE, 12, 13-EpOME, and linoleic acid. The levels of L-Glutamic acid, Phenylalanine, Hippuric acid, LysoPC (18:0), LysoPC (18:1(9Z)), and Alpha-linolenic acid was significantly decreased. However, the levels of these metabolites in YL were reversed and returned to normal or near-normal levels compared to MG. Therefore, they are considered potential biomarkers for the hypolipidemic effects of YL. Figure 6 shows the trend of biomarker levels in the different groups, which indicates a significant change in the potential biomarkers between CG and MG, and these changes can be reversed by YL treatment.

### 3.5 Effect of YL on inflammation and angiogenesis

**FIGURE 7:**
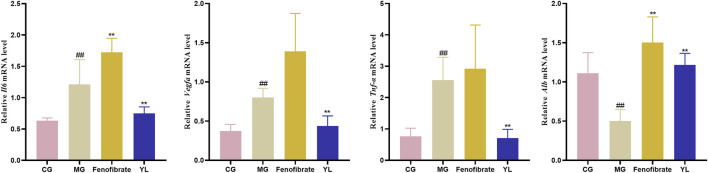
The expression of *Il6*, *Vegfa*, *Tnf-α* and *Alb* mRNA level. Values are mean ± SD, *n* = 6. ^#^ *p* < 0.05, ^##^ *p* < 0.01 vs. CG; *p < 0.05, ^**^ *p* < 0.01 vs. MG.

As shown in Figure 7, compared with the CG, the levels of *Il6*, *Vegfa*, and *Tnf-α* mRNA significantly increased in the MG (*p* < 0.01), whereas the *Alb* mRNA was significantly decreased (*p* < 0.01). As expected, YL administration significantly enhanced the expression of *Alb* mRNA and declined *Il6*, *Tnf-α*, and *Vegfa* mRNA expression compared with those in the MG (all *p* < 0.01).

### 3.6 Correlation analysis

**FIGURE 8:**
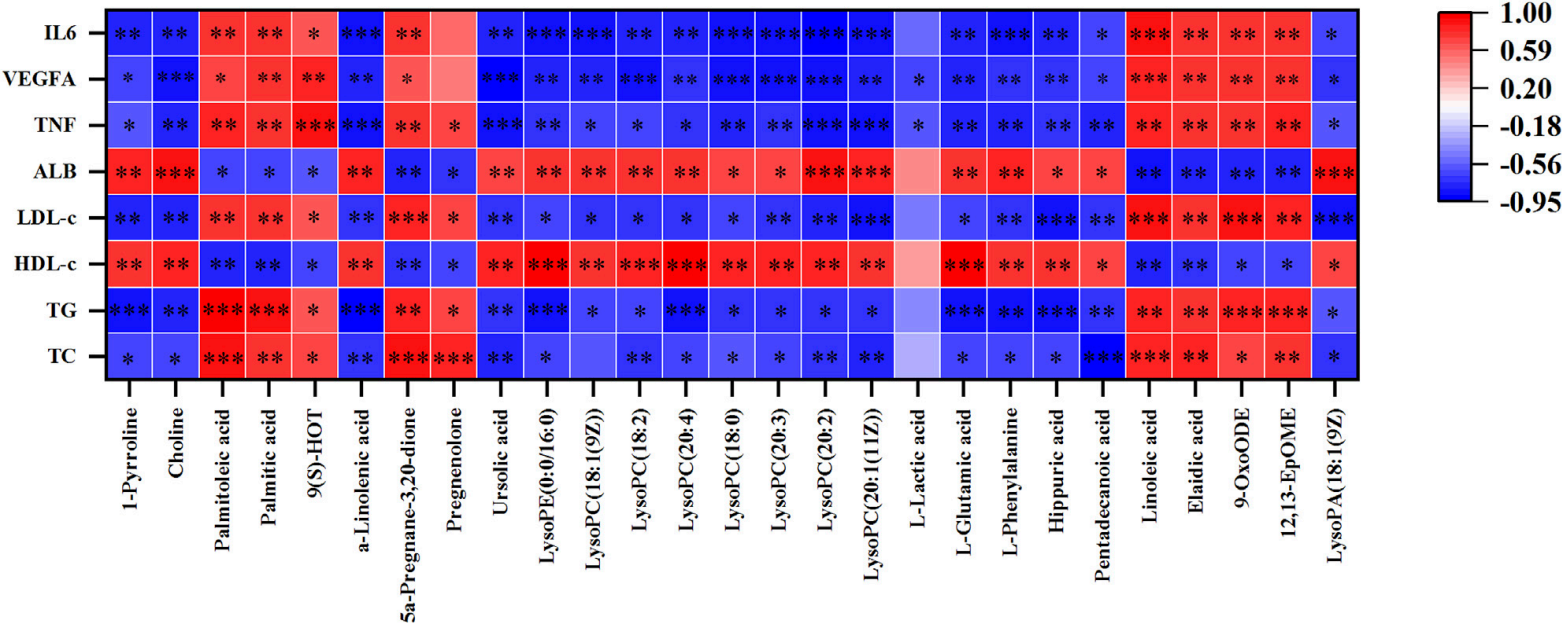
The Spearman’s correlation analysis of metabolic parameters and metabolites. Correlations of 27 altered metabolites and 8 metabolic parameters in MG and YL with spearman correlation analysis.

**FIGURE 9:**
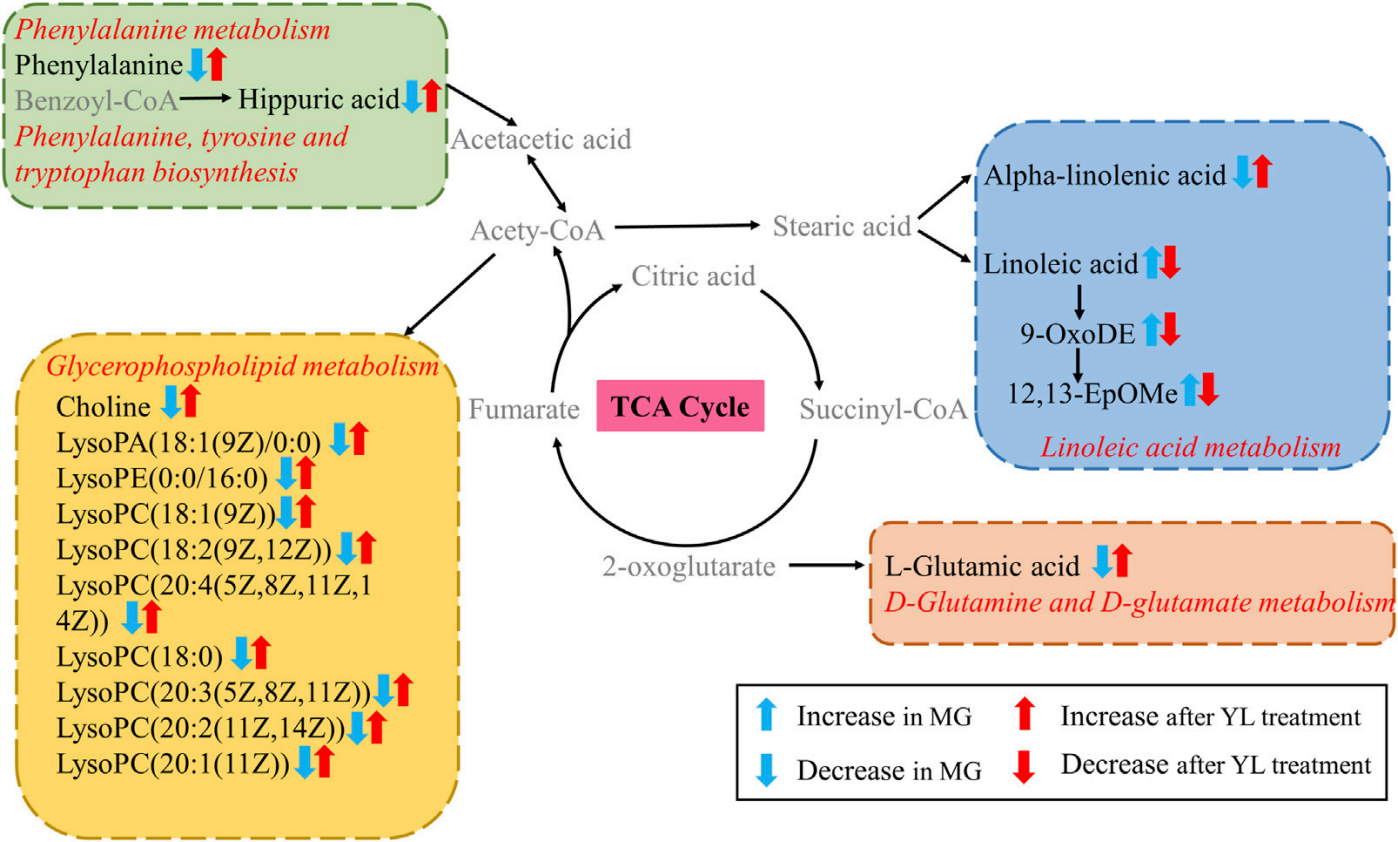
Metabolic network related to hyperlipidemia based on KEGG. Metabolite names in gray were not detected in present study.

To further investigate the relationship between metabolites, metabolic parameters, and their regulatory effects (including dyslipidemia, inflammation, and angiogenesis), we analyzed the correlation between 27 altered metabolites and 8 metabolic parameters in the hyperlipidemia and YL groups with Spearman correlation analysis. The result suggested that the altered metabolites of amino acid metabolites (L-glutamic acid, L-phenylalanine, hippuric acid, etc.), fatty acid metabolites (linoleic acid, Alpha-Linolenic acid, 9-OxoODE, etc.), phospholipids metabolites (LysoPC (18:1(9Z)), LysoPC (18:0), etc.) were significantly associated with blood lipid levels in Figure 8. Positive and negative correlations are represented by red and blue colors, respectively. Furthermore, the stronger the link between the two biomarkers, the deeper the hue (Wang et al., 2022). According to the KEGG database, we graphed the metabolic network associated with the Triton WR-1339-induced hyperlipidemia model and the relevant biomarkers affected by YL (Figure 9). The results implied that YL promoted its anti-hyperlipidemia effect by improving the levels of endogenous metabolites, and anti-inflammatory and modulating angiogenic capacity.

This study illuminated that the intervention of YL could ameliorate dyslipidemia and abnormal metabolites in triton 1339-WR-induced hyperlipidemia mice. The pathogenesis of hyperlipidemia is mainly associated with disorders of metabolic pathways including linoleic acid metabolism, glycerophospholipid metabolism, phenylalanine metabolism, phenylalanine, tyrosine and tryptophan biosynthesis, and D-glutamine and D-glutamate metabolism.

## 4 Discussion

Hyperlipidemia is characterized by abnormal lipid levels in the blood and constitutes a potentially harmful disease leading to CVDs with high mortality (Ge et al., 2022). Previous studies found that YL may treat hyperlipidemia by modulating angiogenesis and suppressing inflammatory responses (Chen et al., 2019; Xiao et al., 2023a). Therefore, we investigated the hypolipidemic effects of YL in hyperlipidemia mice to understand whether the YL could reverse triton WR-1339-induced dyslipidemia and metabolic abnormalities and exert anti-inflammatory and angiogenic effects, as well as to observe the changes of metabolites in mice to illuminate the underlying mechanism of hyperlipidemia alleviation. It is critical to investigate the occurrence and progression of lipid metabolism disorder using serum as a research object and metabolomics technology. Our results identified 27 serum biomarkers associated with hyperlipidemia, primarily phospholipids, fatty acids, and amino acid metabolites. Meanwhile, serum lipid levels were significantly improved after YL treatment, which demonstrated the lipid-lowering effect of YL.

Hyperlipidemia is diagnosed in clinical practice by evaluating four lipid indices showing elevated levels of TC, TG, and LDL-c and declined levels of HDL-c (Ta et al., 2023). Our results showed that TC, TG, and LDL-c levels were significantly elevated and HDL-C levels declined in Triton WR-1339-induced hyperlipidemia mice compared with CG, demonstrating that the hyperlipidemia model was successfully established. However, the levels of TC, TG, and LDL-c were significantly decreased and the levels of HDL-c were significantly increased in the YL compared with the MG. These results proved the lipid-lowering effect of YL.

The most obvious manifestation of the development of hyperlipidemia is the disorder of lipid metabolism. Evidence is growing that hyperlipidemia is involved with phospholipids and fatty acids (Li et al., 2016; Sun et al., 2017; Ren et al., 2023). We have determined some metabolites related to lipid metabolism in the serum of mice treated with YL by serum metabolomics analysis. The results of the clustering analysis showed that the major differential metabolite pathways in the different groups were linoleic acid metabolism, glycerophospholipid metabolism, phenylalanine metabolism, phenylalanine, tyrosine and tryptophan biosynthesis, and glutamine and glutamate metabolism. Alpha-linolenic acid has been shown in studies to dramatically increase insulin sensitivity and anti-inflammatory state, which is crucial for the prevention of CVDs (Zhang et al., 2016; Yue et al., 2021; Ren et al., 2023). The alpha-linolenic acid diet has also been reported to improve lipid profiles by lowering TG, TC, and LDL levels in patients with hyperlipidemia or hyperglycemia. Furthermore, alpha-linolenic acid dramatically lowered liver weight, hepatic cholesterol levels, and the expression of cholesterol synthase enzymes linked with hyperlipidemia, and it may reduce plasma and liver cholesterol content via modulating RCT (Andersen and Fernandez, 2013; O'Reilly et al., 2020). Conjugated linoleic acids are critical for fat deposition in the liver as well as for the development and improvement of IR (Moon et al., 2009). In addition, studies have indicated that unsaturated fatty acids reduce the incidence of hyperlipidemia, whereas saturated fatty acids have the opposite effect (Ito, 2015; Jenkins et al., 2015; Ren et al., 2023). As downstream products of linoleic acid, 9-OxoDE and 12,13-EpOMe were significantly increased in the serum of hyperlipidemic mice. After the treatment of YL, the lipid-related metabolites in the serum of hyperlipidemic mice could be reversed, and the disordered lipid metabolism could be improved, which could play a positive role in hyperlipidemia.

The basic structural components of cell membranes are glycerophospholipids and sphingolipids, and Lysophosphatidylcholine (lysoPCs) is generated by the hydrolysis of oxidized phosphatidylcholine in LDL by phospholipase A2 and act in a range of biological processes (Ren et al., 2023; Zhao et al., 2023). Phosphatidylcholine (PC), phosphatidylglycerol (PG), and phosphatidylethanolamine (PE) are the three types of glycerophospholipids (Rowlett et al., 2017). LysoPCs behave as a direct response molecule, capable of inducing inflammation, cyclooxygenase production, and autoimmune reactions either independently or by activating specific G protein-coupled receptors (Brkic et al., 2012). Furthermore, lysoPCs are the primary metabolic intermediates of glycerophospholipid metabolism, and lysoPC has been linked to CVDs such as atherosclerosis and hyperlipidemia, with a positive association between hyperlipidemia and glycerophospholipid metabolism problems (Miao et al., 2015; Zhao et al., 2015; Paapstel et al., 2018). YL may perform a critical role in ameliorating disorders of glycerol and phospholipid metabolism, resulting in reduced inflammation and suppression of hyperlipidemia development. Choline is involved in the lipid metabolism pathway of the body, and mice lacking the PEMT gene, which encodes the function of liver choline synthesis, have a large accumulation of fatty fat in the liver, resulting in fatty liver, suggesting that choline plays an essential role in the regulation of lipid metabolism (Song et al., 2005). In the present study, a series of lysophospholipids were significantly decreased in the MG compared with CG and recovered after YL treatment. There is growing evidence that lysophospholipids are implicated in energy metabolism, inflammation, and endothelial damage (Yan et al., 2020). However, the exact mechanisms are still too complex and need to be explored in greater depth.

In addition to glycerophosphate metabolism, changes in amino acid levels can also affect lipid levels. Some studies have shown that amino acids, which are primarily involved in numerous metabolic pathways such as the TCA cycle, gluconeogenesis, and others, play an essential role in metabolism (Wu, 2009; Park and Han, 2018). Through the TCA cycle, glutamic acid can be transformed into aspartic acid, and glutamine synthetase converts glutamate into glutamine, which is crucial for the glycolysis of energy metabolism and lipid metabolism in the body (Davalli et al., 2012). Phenylalanine is a nutritional precursor of metabolites generated by the gut microbiota and is clinically and mechanistically associated with CVDs and the resulting major adverse cardiovascular events through the action of adrenergic receptors (Nemet et al., 2020; Zhang et al., 2022). Phenylalanine is an amino acid precursor to tyrosine, which is biologically transformed into L-tyrosine. Tyrosine is also a precursor of catecholamines, which facilitate lipid metabolism and may be a biomarker of hyperlipidemia (Zhang et al., 2009; Zhang et al., 2022). In the present study, the serum levels of glutamate and phenylalanine were elevated in YL-treated mice, indicating that the favorable effects of YL may be attributed to the regulation of phenylalanine metabolism and phenylalanine, tyrosine and tryptophan biosynthesis under hyperlipidemia conditions.

Inflammation, combined with the occurrence and progression of hyperlipidemia, can hasten fat accumulation in liver cells, whereas massive fat creation constantly exacerbates inflammation, resulting in increased blood lipids (Tietge, 2014). Furthermore, YL may treat hyperlipidemia by modulating inflammatory and angiogenesis mechanisms (Xiao et al., 2023a). We found that the expression of *Il6*, *Vegfa*, and *Tnf-α* mRNA were significantly enhanced (*p* < 0.01), and the expression of *Alb* mRNA (*p* < 0.01) was significantly reduced in the MG compared with those in the CG. After treatment with YL, the expression of *Il6*, *Vegfa*, and *Tnf-α* mRNA (*p* < 0.01) were inhibited and promoted the expression of *Alb* mRNA (*p* < 0.01). Therefore, the effects of YL can effectively ameliorate hyperlipidemia by regulating angiogenesis and anti-inflammatory mechanisms. Besides, in connection with the correlation analysis, YL may synergistically affect amino acid, phospholipid, and fatty acid levels and regulate lipid levels in hyperlipidemia mice through metabolic pathways such as linoleic acid metabolism, glycerophospholipid metabolism, phenylalanine metabolism, phenylalanine, tyrosine and tryptophan biosynthesis, and glutamine and glutamate metabolism.

In summary, YL can significantly reverse the triton WR-1339-induced abnormal levels of the metabolites linoleic acid, 12,13-EpOME, lysoPC(18:1(9Z)), lysoPC(18:0), choline, L-phenylalanine, and L-glutamic acid, and thereby interfere with the signaling pathways of linoleic acid metabolism, glycerophospholipid metabolism, phenylalanine metabolism, phenylalanine, tyrosine and tryptophan biosynthesis, and glutamine and glutamate metabolism, resulting in effective treatment of hyperlipidemia. However, the exact mechanism is far too complex, and further study is needed to validate the levels of the target gene or protein expression associated with the altered pathway and demonstrate how YL can lipid-lowering at the molecular level. Furthermore, further studies are also required to analyze the active constituents of YL and its relationship with key metabolites and lipid metabolism parameters, and to identify the biological activity and mechanism of action.

## 5 Conclusion

YL could effectively ameliorate the hyperlipidemia resulting from triton WR-1339-induced. The beneficial actions of YL have been primarily associated with the correction of metabolic disorders and the maintenance of the dynamic balance of metabolites, which mainly involve linoleic acid metabolism, glycerophospholipid metabolism, phenylalanine metabolism, phenylalanine, tyrosine and tryptophan biosynthesis, and glutamine and glutamate metabolism pathways. The current study suggests that YL can be used as a TCM prescription for the treatment of hyperlipidemia, and the results might provide novel insights into the lipid-lowering effect of YL, and expand the comprehension of the relationship between metabolites and lipid-lowering effects, as well as provide new scientific evidence for clinical application and promotion of YL.

## Data Availability

The original contributions presented in the study are included in the article/Supplementary Material, further inquiries can be directed to the corresponding authors.
