# Supplementary material for: Metabolomics analysis delineates the therapeutic effects of Yinlan Tiaozhi capsule on triton WR-1339 -induced hyperlipidemia in mice
Source: Front Pharmacol. 2023 Oct 30;14:1252146. doi: 10.3389/fphar.2023.1252146 (PMC10642944; doi:10.3389/fphar.2023.1252146)
Supplement: Supplementary file 1 [file DataSheet1.docx]

**Supplementary Information**


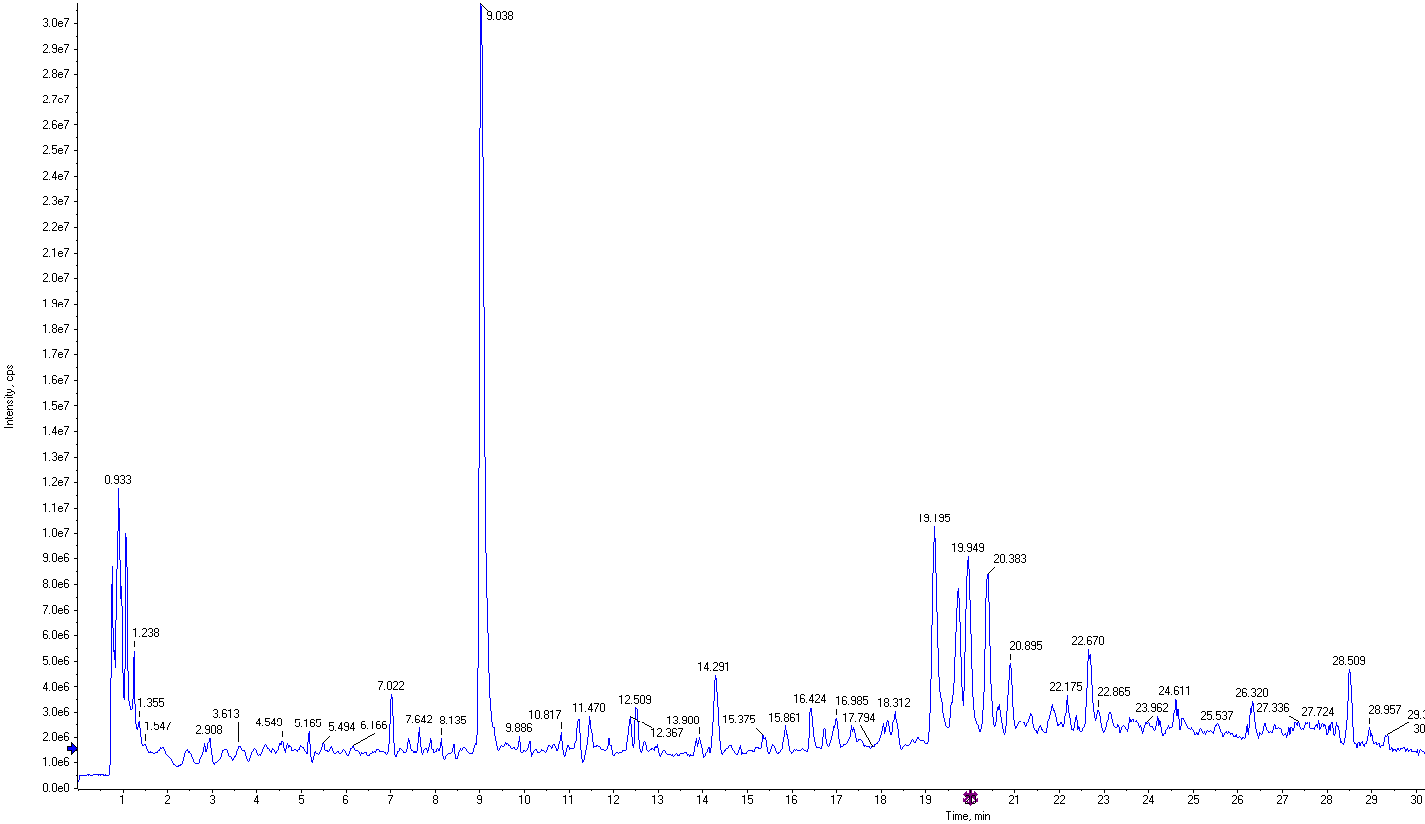
Yinlan Tiaozhi capsules were first broken into powder. Weigh 0.25 g in a 50 mL stoppered conical flask, add 25 mL of 50% ethanol, ultrasonic treatment for 30 min, cool, shake well, centrifuge at 13 000 r/min for 10 min, take the supernatant and filter through 0.22 μm microporous membrane. The UPLC analysis was carried out on a SHIMADZU ExionLC system (Shimadzu, Japan). The chromatographic separations were achieved on a Waters UPLC BEH C18 column (2.1 mm×100 mm, 1.7μm), flow rate 0.3mL·min-1, injector temperature 4℃, column temperature 35℃, injection volume 1μL. Mobile phase: gradient elution of acetonitrile (A) -0.1% formic acid water (B) (0-3 min, 8%A-25; 3-12 min, 25%-47%A; 12-25 min, 47%-79%A; 25-30 min, 79%-95%A). The high-resolution mass detection was performed on an AB SCIEX X500R QTOF-MS/MS system (Sciex, United States). MS was performed both in positive and negative ion modes with electrospray ionization (ESI). The optimization source parameters were set as follows: ion voltage: -4500 V and +5500 V, Gas1: 55 psi; Gas2: 55 psi; curtain gas: 35 psi; de-clustering potential voltage: 60 V; ion source temperature: 500 °C; collision energy: 35 V; collision energy spread: 15 V; full scan: m/z 50-1500. A specific UPLC-QTOF-MS method was used to identify the chemicals in YL. The TIC chromatograms of YL substance basis in negative and positive ion modes as shown in Figure S1A, a total of 11 compounds were identified by comparing their retention time with that of reference compounds or comparing their retention behaviors and proposed fragments with that in literature. The extracted ion chromatogram of reference compounds as shown in Figure S1B.

**a**

**A**

**b**


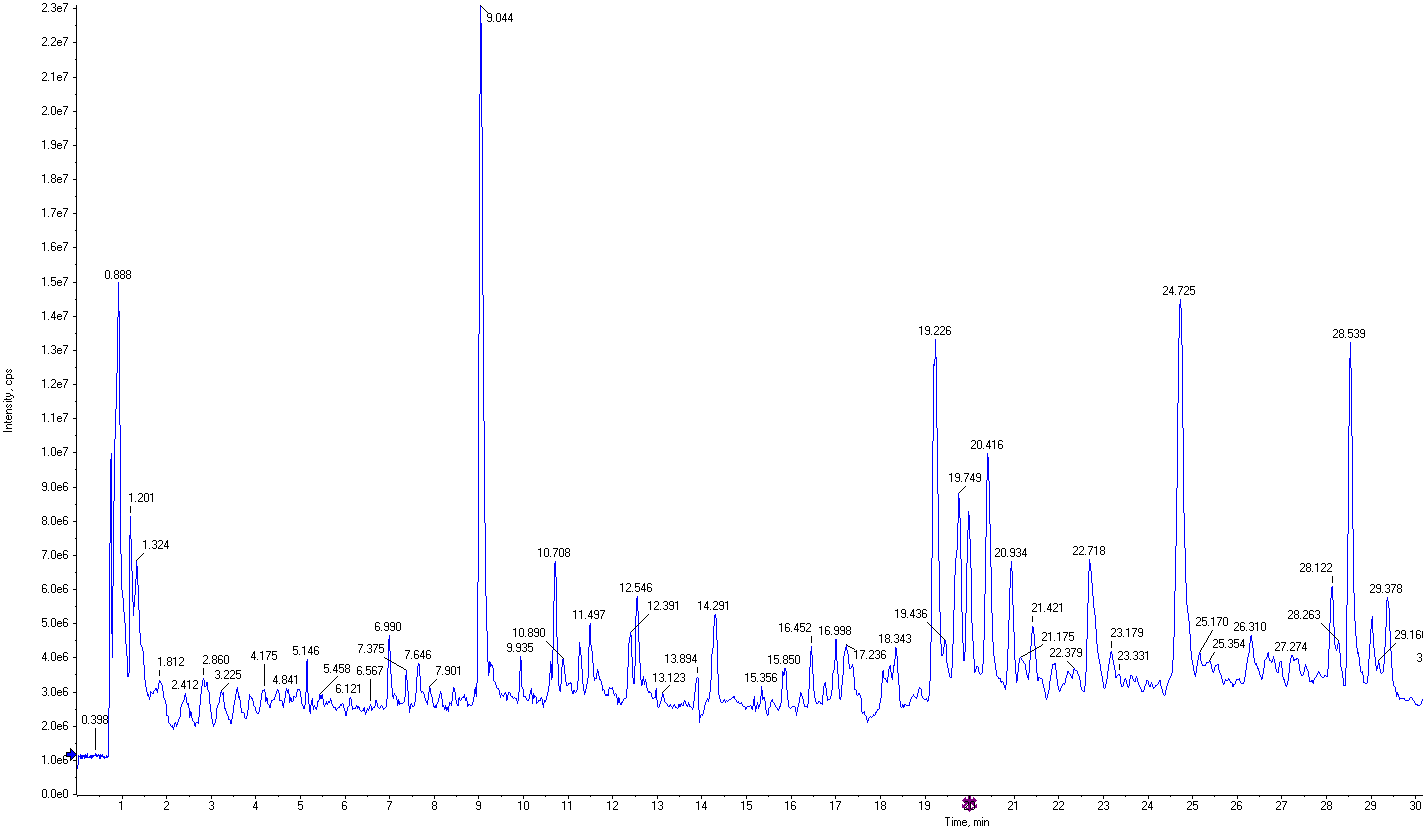


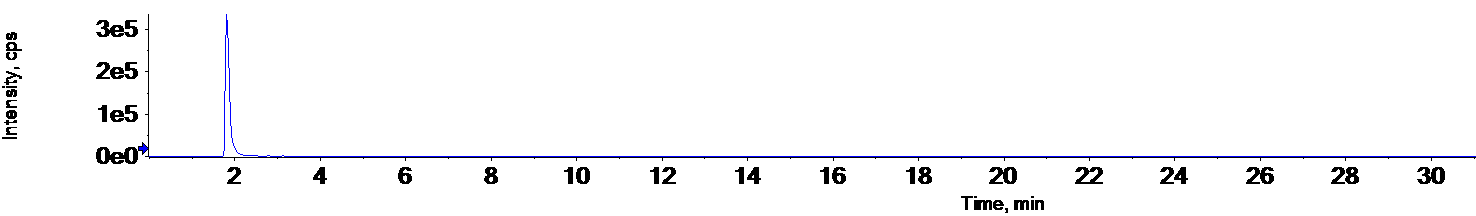


**B**

**c**


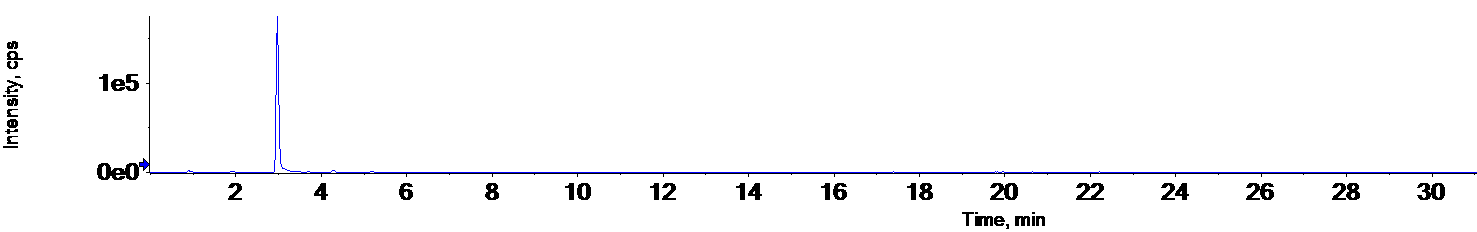


**d**


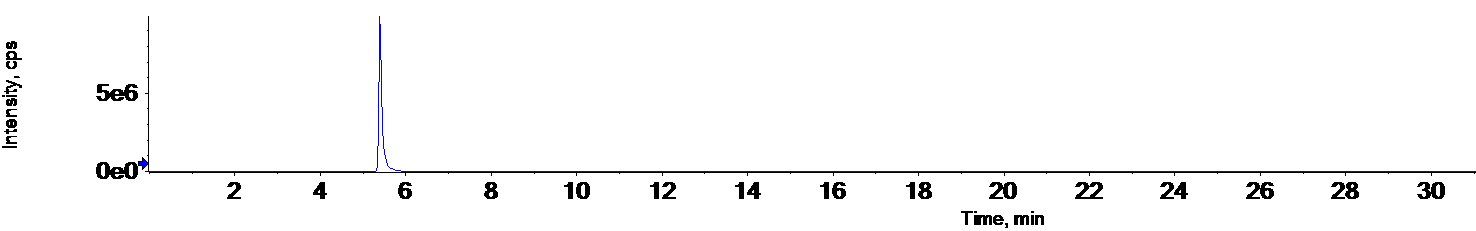


**f**

**e**


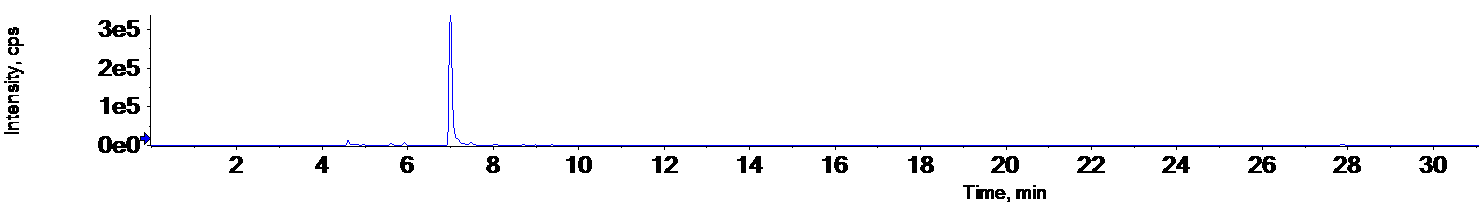


**g**


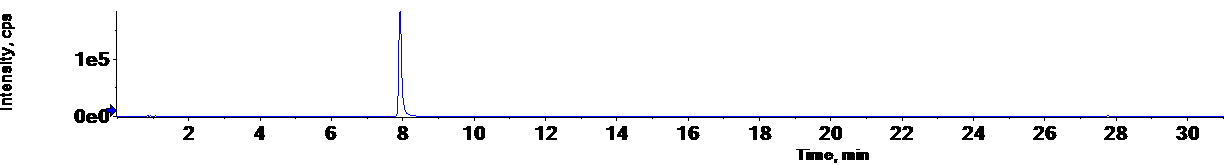


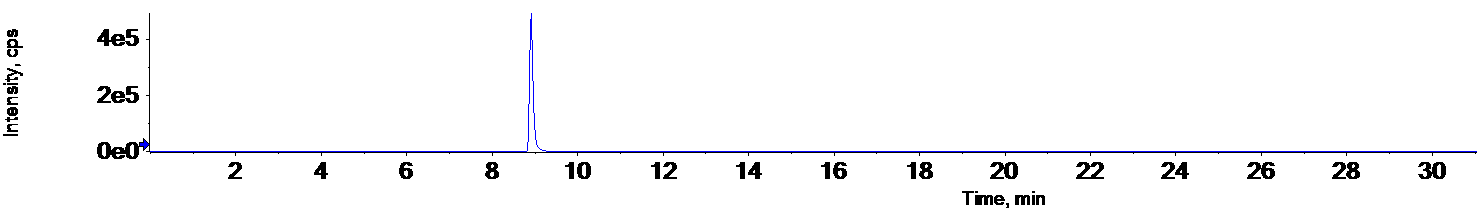


**h**


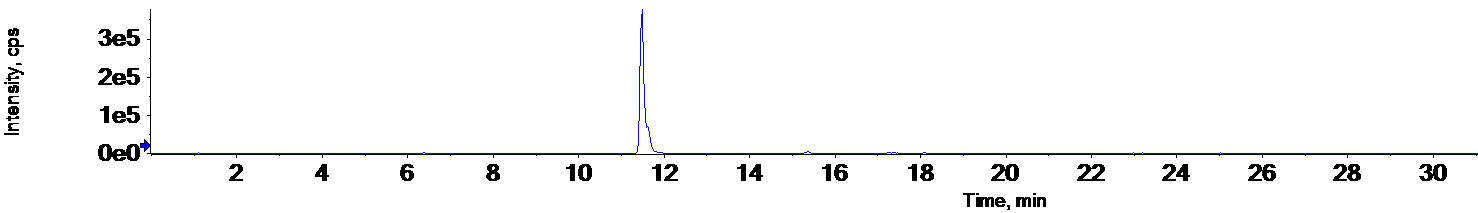


**i**


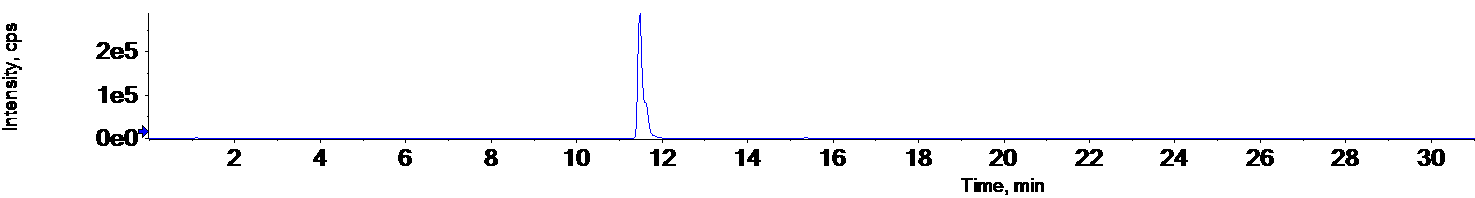


**j**


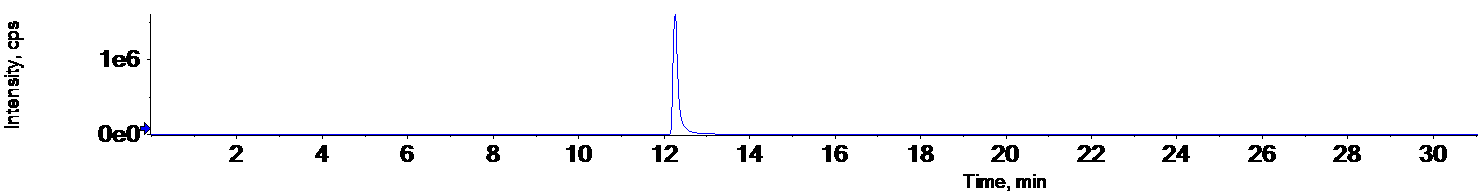


**k**


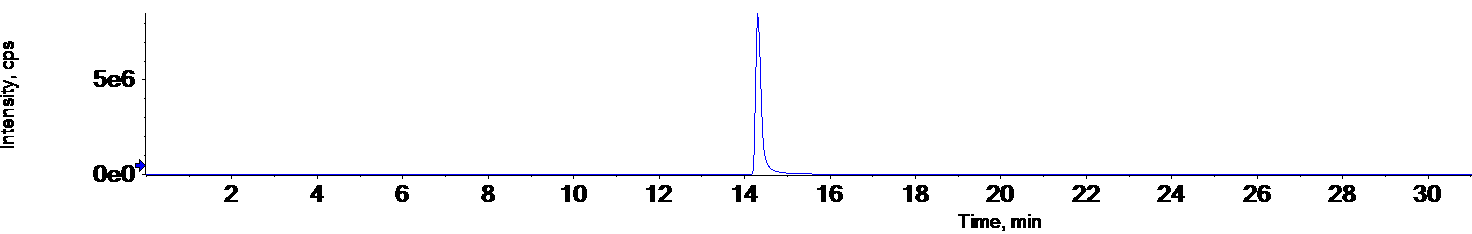


**l**


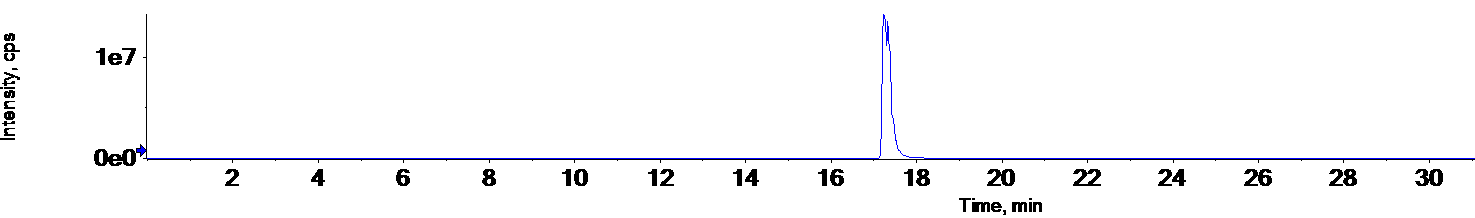


**m**


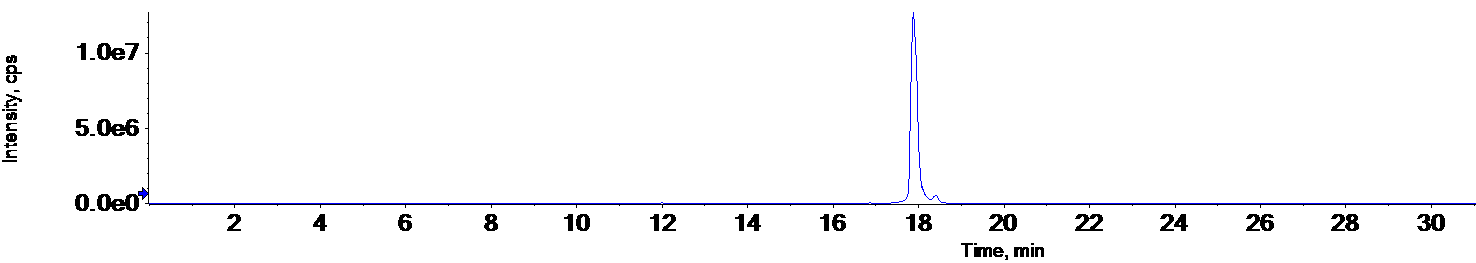


**n**


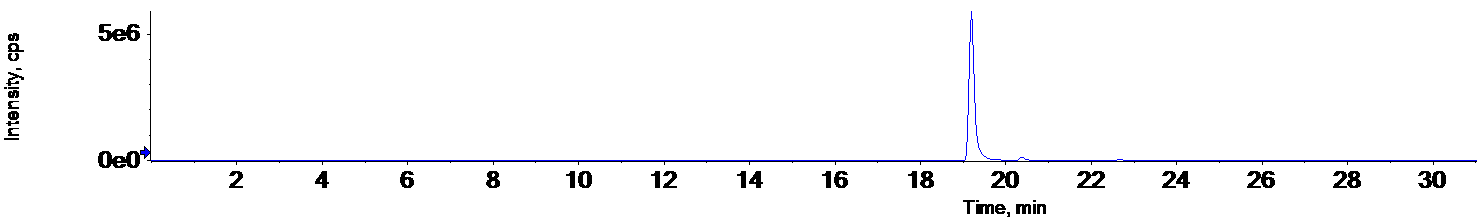


**p**

**o**


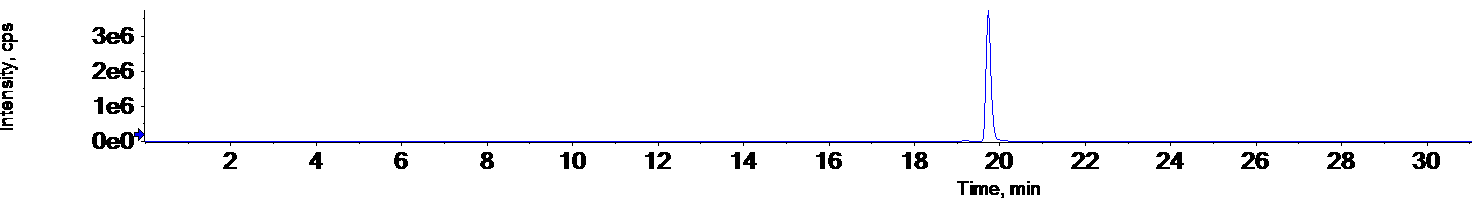


Figure S1. Chemical profile of YL by UPLC-QTOF-MS/MS. (A) TIC chromatograms of YL substance basis in negative (a) and positive (b) ion modes. (B) The extracted ion chromatogram of reference compounds. c. Gallic acid d. Protocatechuic acid e. Vicenin-2 f. Rutin g. Ferulic acid h. Naringin i. Ginkgolide A j. Ginkgolide B k. Luteolin l. Naringenin m. Isomeranzin n. Limonin o. Chrysin p. Pinocembrin
